# PTEN inhibits BMI1 function independently of its phosphatase activity

**DOI:** 10.1186/1476-4598-8-98

**Published:** 2009-11-10

**Authors:** Catherine Fan, Lizhi He, Anil Kapoor, Adrian P Rybak, Jason De Melo, Jean-Claude Cutz, Damu Tang

**Affiliations:** 1 Division of Nephrology, Department of Medicine, McMaster University, McMaster University, Hamilton, ON, Canada; 2 Father Sean O'Sullivan Research Institute, St Joseph's Hospital, Hamilton, ON, Canada; 3 The Hamilton Centre for Kidney Research (HCKR), St Joseph's Hospital, Hamilton, ON, Canada; 4 Department of Surgery, McMaster University, Hamilton, ON, Canada; 5 Department of Pathology and Molecular Medicine, McMaster University, Hamilton, ON, Canada

## Abstract

**Background:**

*PTEN *is the second most mutated tumor suppressor gene other than p53. It suppresses tumorigenesis by dephosphorylating phosphatidylinositol (3,4,5)-triphosphate (**PIP3**) to phosphatidylinositol (4,5)-biphosphate (**PIP2**), thereby directly inhibiting phosphatidylinositol 3 kinase (**PI3K**)-mediated tumorigenic activities. Consistent with this model of action, cytosolic PTEN is recruited to the plasma membrane to dephosphorylate PIP3. While nuclear PTEN has been shown to suppress tumorigenesis by governing genome integrity, additional mechanisms may also contribute to nuclear PTEN-mediated tumor suppression. The nuclear protein BMI1 promotes stem cell self-renewal and tumorigenesis and PTEN inhibits these events, suggesting that PTEN may suppress BMI1 function.

**Results:**

We investigated whether PTEN inhibits BMI1 function during prostate tumorigenesis. PTEN binds to BMI1 exclusively in the nucleus. This interaction does not require PTEN's phosphatase activity, as phosphatase-deficient PTEN mutants, PTEN/C124S (CS), PTEN/G129E (GE), and a C-terminal PTEN fragment (C-PTEN) excluding the catalytic domain, all associate with BMI1. Furthermore, the residues 186-286 of C-PTEN are sufficient for binding to BMI1. This interaction reduces BMI1's function. BMI1 enhances hTERT activity and reduces p16^INK4A ^and p14^ARF ^expression. These effects were attenuated by PTEN, PTEN(CS), PTEN(GE), and C-PTEN. Furthermore, knockdown of PTEN in DU145 cells increased hTERT promoter activity, which was reversed when BMI1 was concomitantly knocked-down, indicating that PTEN reduces hTERT promoter activity via inhibiting BMI1 function. Conversely, BMI1 reduces PTEN's ability to inhibit AKT activation, which can be attributed to its interaction with PTEN in the nucleus, making PTEN unavailable to dephosphorylate membrane-bound PIP3. Furthermore, BMI1 appears to co-localize with PTEN more frequently in clinical prostate tissue samples from patients diagnosed with PIN (prostatic intraepithelial neoplasia) and carcinoma compared to normal prostate epithelium. While PTEN co-localized with BMI1 in 2.4% of normal prostate epithelial cells, co-localization was observed in 37.6% and 18.5% of cells in PIN and carcinoma, respectively. Collectively, we demonstrate that PTEN inhibits BMI1 function via binding to BMI1 in a phosphatase independent manner.

**Conclusion:**

We demonstrate that nuclear PTEN reduces BMI1 function independently of its phosphatase activity. It was recently observed that nuclear PTEN also suppresses tumorigenesis. Our results, therefore, provide a plausible mechanism by which nuclear PTEN prevents tumorigenesis.

## Introduction

The polycomb group (PcG) *BMI1 *gene maintains the proliferation potential and self-renewal of hematopoietic and neural stem cells [1,2]. This is in part attributable to BMI1-mediated suppression of p16^INK4A^, p19^ARF^/p14^ARF^, and E4F1 [3-6]. This developmental function of BMI1 is in line with its oncogenic role in leukemia. The *BMI1 *gene was initially isolated as an oncogene which cooperated with c-Myc in retrovirus-induced B and T cell leukemia [7,8]. Overexpression of *BMI1 *transformed lymphocytes [9,10] and was detected in 25% of mantle cell lymphomas [11]. BMI1 is positively associated with unfavorable prognosis in patients with diffuse large B cell lymphomas and myelodysplastic syndrome [12,13]. Increases in BMI1 were also reported in epithelial malignancies, including non-small cell lung cancer (NSCLC) [14], colon cancer [15], breast cancer [16], and nasopharyngeal carcinoma [17].

BMI1 may also promote prostate tumorigenesis. Increases in *BMI1 *mRNA were detected in prostate cancer cell lines, xenografts and human primary prostate carcinomas, as well as primary prostate tumors derived from the TRAMP transgenic mouse model [18]. Prostate cancer patients with an 11-gene signature, which is associated with BMI1 expression, are more likely to have an unfavorable prognosis when compared to those without this signature [18]. Additionally, metastatic prostate carcinoma precursor cells that are double-positive for BMI1 and another polycomb-group protein EZH2 are more tumorigenic than those which are negative for both proteins [19].

Mechanistically, BMI1 promotes tumorigenesis, at least in part, via inhibiting p16^INK4A ^and p19^ARF ^expression, and enhancing human telomerase reverse transcriptase (hTERT) activity [17,20], leading to a bypass of senescence. *BMI1*^-/- ^hematopoietic progenitors express increased levels of p16^INK4A ^and p19^ARF^, and accumulate high levels of the senescence marker SA-β-Gal [5]. *BMI1*^-/- ^mouse embryonic fibroblasts (MEFs) undergo premature senescence [21] and overexpression of *BMI1 *in MEFs and human fibroblasts extends their replicative life spans [21,22]. Consistent with these observations, BMI1 immortalizes human nasopharyngeal and mammary epithelial cells [17,20]. However, how BMI1 is regulated during tumorigenesis remains to be determined.

*PTEN *is a tumor suppressor gene that is frequently mutated in human cancers. This is at least in part attributable to PTEN's action in inhibiting PI3K. PTEN dephosphorylates the 3-position phosphate from the inositol ring of phosphatidylinositol (3,4,5)-triphosphate (**PIP3**) [23], thereby directly inhibiting phosphatidylinositol 3 kinase (**PI3K**)-mediated tumorigenic activities. While PTEN-mediated suppression of the PI3K/AKT pathway is well established, accumulating evidence suggests that nuclear PTEN also plays a critical role in tumor suppression [23]. Although several mechanisms responsible for nuclear PTEN-mediated tumor suppression have been observed [23] (see Discussion for details), additional mechanisms remain likely.

In this investigation, we provide evidence showing that nuclear PTEN suppresses BMI1 function. PTEN binds to BMI1 in the nucleus of prostate cancer cells and reduces BMI1-mediated suppression of p16^INK4A ^and p14^ARF ^as well as BMI1-mediated enhancement of hTERT. Additionally, PTEN co-localizes with BMI1 more frequently in primary prostate carcinomas compared to normal prostate glands. Our observations are consistent with previous findings showing that while BMI1 maintains the proliferation potential of neural stem cells (NSCs) [2], PTEN inhibits this process [24].

## Materials and methods

### Cell lines and plasmids

DU145, MCF7, and 293T cells were purchased from ATCC, and cultured in MEM (DU145) and DMEM (MCF7and 293T) containing 10% FBS and 1% Penicillin-Streptomycin (Invitrogen). Among the most widely used three human prostate cancer cell lines (LNCaP, PC3, and DU145), only DU145 cells express wild type PTEN and BMI1, and therefore were chosen for this research. Human *BMI1 *cDNA was amplified by RT-PCR from HeLa cells, and subsequently subcloned in pcDNA3 and pBabe retrovirus vectors. pGL3-hTERTmin-Luc reporter plasmid, containing a 59bp region of the hTERT promoter (-208 to -150) which has been shown to display maximal promoter activity [25] was constructed from HeLa cell genomic DNA using routine molecular biology techniques.

### Retroviral Infection

Retroviral infection was performed following our previously published procedure [26,27]. Briefly, a gag-pol expressing vector and an envelope-expressing vector (VSV-G) (Stratagene) were transiently co-transfected with a designed retroviral plasmid into 293T cells. After 48 hours, the virus-containing medium was harvested, filtered through a 0.45 μM filter, and centrifuged at 50,000 g for 90 minutes to concentrate the retrovirus. Following the addition of 10 μg/ml of polybrene (Sigma), the medium was used to infect cells. Infection for pBabe-based constructs was selected in puromycin, while infection for pLHCX-based constructs was selected in hygromycin.

### Collecting primary prostate cancer

Prostate tissue was collected at St. Joseph's Hospital in Hamilton, Ontario, Canada with the approval from the local Ethics Board and with consent from the patients. Tumors were examined and graded by pathologists at the Hospital. 42 primary prostate cancer specimens were collected.

### TRAP (Telomeric Repeat Amplification Protocol) assay

A TRAP kit (TRAPEZE^® ^Telomerase Detection kit) was purchased from Chemicon International. TRAP assay was performed according to the manufacturer's instruction.

### Western blot and immunoprecipitation

Cell lysates were prepared and western blot was performed according to our published procedure [26]. 50 μg protein of total lysate was separated on SDS-PAGE gel and transferred onto Immobilon-P membranes (Millipore). Membranes were blocked with 5% skim milk and then incubated with the indicated antibodies at room temperature for 1 hour. Signals were detected using an ECL Western Blotting Kit (Amersham). Primary antibodies used were: polyclonal anti-BMI1 (1:100, Santa Cruz Biotechnology), polyclonal anti-PTEN (1:100, Upstate Technologies), polyclonal anti-p16^INK4A ^(1:500, Santa Cruz Biotechnology), and polyclonal anti-p14^ARF ^(1:5000, Sigma). Immunoprecipitation of ectopic PTEN and BMI1 was performed by incubation of 200 μg cell lysate protein with specific antibodies plus Protein G agarose (Invitrogen) at 4°C overnight, followed by wash for 6 times in a buffer containing 50 mM Tris (PH 7.5), 100 mM NaCl, 1.5 mM EGTA, 0.1% Triton X-100. The antibodies used for immunoprecipitation were monoclonal anti-PTEN (Santa Cruz Biotechnology), monoclonal anti-FLAG (M2, Sigma) for BMI1 and its mutants, and mouse IgG (Sigma) as a negative control. The immunoprecipitation was analyzed by western blot using polyclonal anti-PTEN (Santa Cruz) and anti-FLAG (Sigma). Immunoprecipitation of the endogenous PTEN-BMI1 complex was carried out by lysing DU145 cell in a HEPES lysate buffer, pH7.0 (20 mM HEPES, 150 mM NaCl, 1 mM EDTA, 1 mM EGTA, 1% Triton X-100, 25 mM sodium pyrophosphate, 1 mM NaF, 1 mM β-glycerophosphate, 0.1 mM sodium orthovanadate, 1 mM PMSF, 2 μg/ml leupeptin and 10 μg/ml aprotinin) containing DSP 2 μM (PIRECE) on ice for 2 hours and quenching free DSP by incubating in 50 mM Tris pH 7.5 on ice for 15 minutes. Immunoprecipitation was then carried out as described above.

### Immunofluorescence

Double immunofluorescence staining was carried out using the following antibodies: monoclonal anti-PTEN (Santa Cruz, 1 μg/ml) or a polyclonal anti-PTEN (1:100; Upstate Technologies), polyclonal anti-FLAG or a monoclonal anti-FLAG (M2, 1:500; Sigma), FITC-Donkey anti-mouse IgG (1:200; Jackson Immuno Research) and Rhodamine-Donkey anti-rabbit IgG (1:200; Jackson Immuno Research) were used as secondary antibodies. Images were captured using Axiovert 200 M confocal microscope and AxioVision 3 software.

For double immunofluorescence staining prostate tissues, tissues were deparaffinized, rehydrated, and subjected to antigen-retrieval and endogenous peroxidase-quenching. Tissue sections were blocked for 1 hour at room temperature in 3% donkey serum and 3% BSA in TBST. Dual-IF staining was carried out using a TSA Plus kit (PerkinElmer) according to the manufacturer's protocol. Sections were counterstained with DAPI and digital images were processed as described above.

### Statistical analysis

Analysis was performed using Northern Eclipse 4.0 software (manual cell counter) for Windows. Approximately 1000 cells from randomly selected fields were counted for each normal, PIN, and cancer foci per patient. Mean percentages of positively-stained cells were then analyzed using GraphPad 4.0 for Windows.

## Results

### BMI1 interacts with PTEN

**Figure 1:**
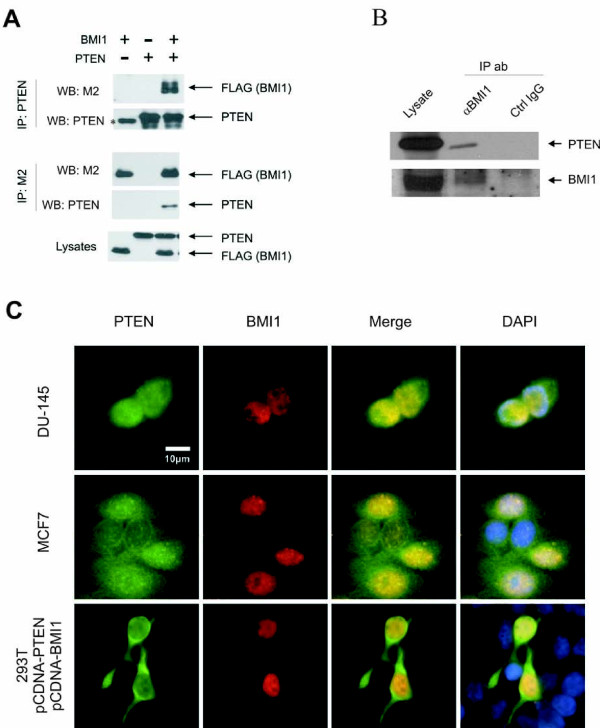
**PTEN binds to BMI1**. (**A**) 293T cells were transiently transfected with FLAG-tagged *BMI1 *and HA-tagged *PTEN*. Cell lysates were prepared and immunoprecipitated with anti-PTEN (top panel) and anti-FLAG (M2) (middle panel) antibodies. The precipitates and lysates (bottom panel) were analyzed by western blot using the indicated antibodies. The * symbol indicates endogenous PTEN. (Note: the reason why endogenous PTEN was not detected in the lysate panel was attributable to a low level of endogenous PTEN in 293T cells). (**B**) DU145 cell lysates were cross-linked with DSP, immunoprecipitated with anti-BMI1 antibody or control IgG, and analyzed by western blot for PTEN and BMI1. Twenty percent of cell lysate used for immunoprecipitation was also analyzed by western blot. (**C**) Co-localization between PTEN and BMI1. Ectopic PTEN and ectopic BMI1 in 293T cells and their respective endogenous proteins in MCF-7 and DU145 cells were examined by double immunofluorescent (IF) staining. Nuclei were counter-stained with DAPI (blue). Scale bar represents 10 μM.

BMI1 determines the proliferation potential of neural stem cells (NSCs) [2], a process that is inhibited by PTEN [24]. Additionally, while BMI1 promotes tumorigenesis in a variety of human cancers [28], PTEN potently suppresses tumorigenesis [29,30]. These observations suggest that PTEN may negatively regulate BMI1 function. Since both BMI1 [7,8] and PTEN reside in the nucleus [31], we hypothesized that BMI1 may associate with PTEN. When transiently co-expressed in 293T cells, a complex containing both BMI1 and PTEN could be immunoprecipitated via either BMI1 or PTEN (Fig 1A), while control IgG did not precipitate either protein (data not shown). This association was also detected between endogenous BMI1 and endogenous PTEN (Fig 1B).

To further examine this interaction, we determined whether PTEN co-localizes with BMI1 inside the cell. When ectopically expressed, PTEN and BMI1 were co-localized in the nucleus (Fig 1C, the 293T cell panel). Furthermore, endogenous BMI1, which was stained in a "punctuate" manner in the nuclei of DU145 and MCF7 cells, co-localized with endogenous nuclear PTEN (Fig 1C). Both anti-BMI1 and anti-PTEN antibodies specifically recognized their respective proteins. The anti-BMI1 antibody did not produce any detectable signals in BMI1-negative LNCaP cells in western blot and in IF procedures (see our recent publication) [32]. The anti-PTEN antibody produced no-detectable signals in PTEN-null LNCaP and U87 cells as well as the signal level was significantly reduced in PTEN siRNA treated DU145 cells in IF procedures (data not shown). We observed that approximately 20% of cells expressed BMI1 at a given time point, a typical expression pattern observed with other polycomb proteins [33]. However, in all BMI1-expressing cells, PTEN co-localized with BMI1 in the nucleus. Taken together, the above observations demonstrate that PTEN associates with BMI1 in the nucleus.

### PTEN interacts with BMI1 independently of its phosphatase activity

**Figure 2:**
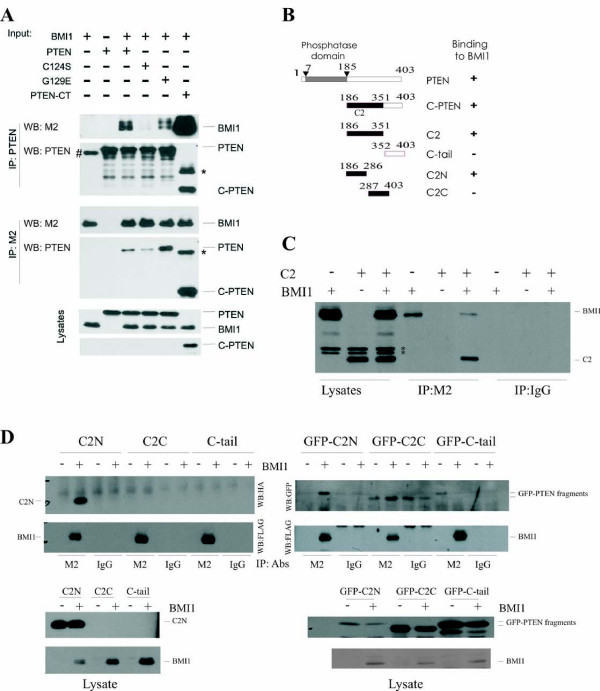
**Characterization of the interaction between PTEN and BMI1 proteins**. (**A**) PTEN binds to BMI1 independently of its phosphatase activity. 293T cells were transiently transfected with *BMI1*, *PTEN, PTEN(C124S) (C124S), PTEN(G129E) (G129E)*, a C-terminal PTEN fragment (residues 186-403) (C-PTEN) for 48 hours. Cell lysates were prepared and immunoprecipitated with anti-PTEN and anti-FLAG (M2) (for ectopic BMI1) antibodies. The precipitates and lysates were analyzed by western blot using the indicated antibodies. The # and * symbols indicate endogenous PTEN and a possible oligomer of C-PTEN, respectively. (**B**) Mapping the BMI1 binding motif of the PTEN protein. A set of PTEN truncation mutants were constructed. Their interaction with BMI1 was examined. C2: C2 domain. The + and - symbols indicate binding or not-binding of individual PTEN proteins to BMI1. (**C**) C2 binds to BMI1. FLAG-tagged *BMI1 *and HA-tagged *C2 *were transfected into 293T cells as indicated. BMI1 was immunoprecipitated with an anti-FLAG antibody (M2) or a control IgG (IgG), followed by western blot examination for BMI1 and C2. 20% of the cell lysates that were used for immunoprecipitations were also analyzed. The * symbols indicate background bands. (**D**) C2N binds to BMI1. *C2N, C2C*, and *C-tail *(left panel) and their GFP fusion counterparts (right panel) were co-transfected with either an empty vector (-) or FLAG-tagged *BMI1 *as indicated, followed by immunoprecipitation with M2 or control IgG (IgG) and then western blot (WB) with the indicated antibodies. The respective cell lysates were shown at the bottom panels.

To characterize the interaction between PTEN and BMI1, we examined whether PTEN's phosphatase activity is required for the association. When ectopically expressed in 293T cells, wild type PTEN, phosphatase-deficient PTEN(C124S) [PTEN(CS)], and PIP3 specific phosphatase-deficient PTEN(G129E) [PTEN(GE)] [34,35] formed a complex with BMI1 as detected by co-immunoprecipitation (Fig 2A). Additionally, a C-terminal fragment of PTEN (C-PTEN) (encompassing residues 186-403, and thus excluding the catalytic domain that lies between residues 1-185) [36] bound to BMI1 (Fig 2A, B). In comparison to PTEN, PTEN(CS) interacted with BMI1 with reduced affinity [Fig 2A, comparing BMI1 co-immunoprecipitated via PTEN with BMI1 co-immunoprecipitated via PTEN(CS) as well as comparing PTEN with PTEN(CS) that were co-immunoprecipitated via BMI1]. This may be attributed to potential conformational changes that might be caused by this mutation rather than due to the lack of phosphatase activity, as C-PTEN binds to BMI1 with increased affinity (Fig 2A). We also examined whether N-PTEN (residues 1-185) interacts with BMI1 and found that N-PTEN was expressed at undetectable levels when co-expressed with BMI1 in 293T cells (data not shown). It was thus difficult to determine if N-PTEN binds to BMI1. Nonetheless, our experiments demonstrate that C-PTEN is sufficient to interact with BMI1 and that PTEN binds to BMI1 independently of its phosphatase activity.

### PTEN binds to BMI1 via residues 186-286

C-PTEN contains two functional domains, the C-2 domain and the C-terminal tail (Fig 2B). To further map the BMI1-binding region, we generated HA-tagged C2 and C-tail PTEN fragments (Fig 2B) and examined their association with BMI1. When co-expressed in 293T cells, immunoprecipitation of FLAG-tagged BMI1 efficiently co-precipitated the C-2 fragment (Fig 2C). We further truncated the C-2 fragment into HA-tagged C2N and C2C (Fig 2B) and co-expressed BMI1 with either C2N or C2C in 293T cells (Fig 2D, bottom-left panel). Immunoprecipitation of BMI1 co-precipitated C2N (Fig 2D, left panel). However, we could not detect C2C and C-tail fragments (Fig 2D, bottom-left panel). This may have been caused by the potential instability of these fragments due to their inability to properly fold. To address this potential issue of instability, we fused the fragments to green fluorescent protein (GFP) and demonstrated their expression in 293T cells (Fig 2D, bottom-right panel). However, these fragments were either immunoprecipitated with control IgG (GFP-C2C) or anti-FLAG (M2) antibody (GFP-C-tail) without the co-expression of FLAG-tagged BMI1 (Fig 2D, top-right panel), demonstrating that these fragments were non-specifically immunoprecipitated. This indicates that the fusion proteins did not fold properly, which is consistent with our inability to detect the expression of C2C and C-trail fragments by western blot (Fig 2D, bottom-left panel).

To further examine C2N-medaited BMI1 binding, we fused C2N to GFP. When co-expressed in 293T cells, GFP-C2N was co-immunoprecipitated via FLAG-tagged BMI1 (Fig 2D, right panel). Taken together, the above experiments reveal that the C2N (186-286) PTEN fragment is sufficient to interact with BMI1.

### PTEN inhibits BMI1 function independently of its phosphatase activity

**Figure 3:**
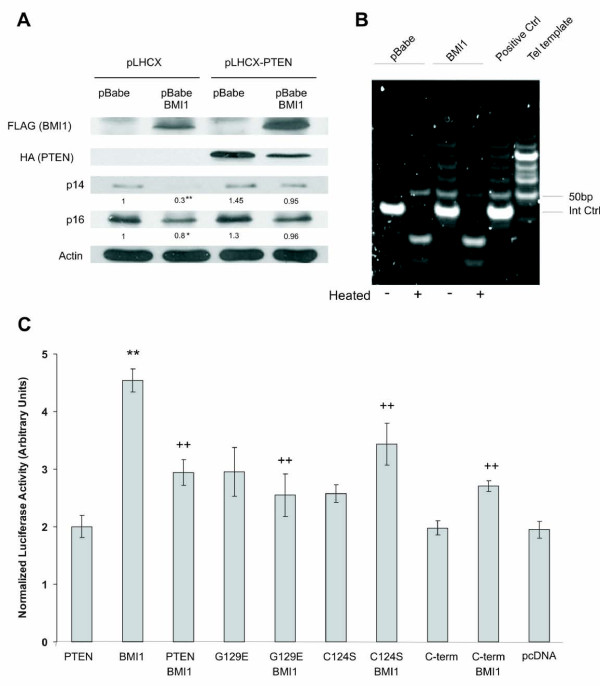
**PTEN inhibits BMI1 function**. (**A**) DU145 cells were stably transfected with pBabe or pBabe-BMI1 retrovirus, followed by transiently transfected with pLHCX (empty vector) and pLHCX-PTEN retrovirus for 48 hours. The expression of FLAG-tagged BMI1, HA-tagged PTEN, p16^INK4A^, p14^ARF^, and actin was examined by western blot using specific antibodies. The relative p14^ARF ^and p16^INK4A ^expression was normalized against the respective actin and then expressed as fold changes of p14^ARF ^and p16^INK4A ^in DU145 cells co-infected with pBabe and pLHCX. The experiment was repeated at least three times by three individuals with identical results and representatives are shown. This information was presented under the p14 and p16 panels. Symbols * and ** show statistical significance (*p *< 0.05 and *p *< 0.01, respectively), in comparison to pBabe/pLHCX infected cells, determined by Student's t-Test (2-tails). (**B**) DU145 cells were stably transfected with pBabe and pBabe-BMI1 retrovirus, followed by assaying for hTERT activity using TRAP assay following the manufacturer's procedure. (**C**) 293T cells were transiently transfected with *PTEN, PTEN(G129E) (G129E), PTEN(C124S) (C124S)*, *C-terminal PTEN fragment *(residues 186-403) (C-term), and BMI1 as indicated together with a hTERT promoter driven luciferase construct plus a β-Gal construct for 48 hours. Luciferase and β-Gal enzymatic activities were determined. Luciferase activities were normalized against β-Gal activities. Each transfection was carried out in triplicate and the experiment was repeated three times. **: *p *< 0.01 (in comparison to pcDNA); ++: *p *< 0.01 (in comparison to BMI1).

Since PTEN and BMI1 function in opposite directions in both stem cell biology and tumorigenesis, the observation that PTEN binds to BMI1 indicates that PTEN may inhibit BMI1 function. BMI1 has been shown to suppress the expression of the *INK4A/ARF *locus, p16^INK4A ^and p14^ARF ^[3,4], which we have also demonstrated recently in prostate cancer cells [32]. To examine whether PTEN affects BMI1-mediated inhibition of p16^INK4A ^and p14^ARF ^expression, we stably expressed BMI1 and PTEN individually and in combination into DU145 cells (Fig 3A). Consistent with previous publications [3,4], ectopic BMI1 reduced endogenous p16^INK4A ^and, to a greater degree, p14^ARF ^expression (Fig 3A). While ectopic PTEN did not enhance p16^INK4A ^or p14^ARF ^expression, it prevented the BMI1-mediated reduction of p16^INK4A ^and p14^ARF ^(Fig 3A).

BMI1 has been shown to increase hTERT activity in mammary epithelial cells [20]. To examine whether BMI1 also up-regulates hTERT activity in prostate cancer, we ectopically expressed BMI1 in DU145 prostate cancer cells using a retrovirus. A TRAP (Telomeric Repeat Amplification Protocol) assay revealed that BMI1 enhanced hTERT activity (Fig 3B). To address the impact of PTEN on BMI1-induced hTERT activation, we co-transfected a BMI1-expressing plasmid along with a hTERT promoter-reporter construct (pGL3-hTERTmin-Luc) [25] with the addition of either an empty vector, PTEN, PTEN(CS), PTEN(GE) or C-PTEN construct into 293T cells (Fig 3C). Consistent with the TRAP assay, BMI1 increased hTERT promoter activity (Fig 3C). Interestingly, PTEN, PTEN(CS), PTEN(GE), and C-PTEN all inhibited the BMI1-mediated activation of the hTERT promoter (Fig 3C). This is consistent with the observation that all these PTEN proteins interact with BMI1 (Fig 2A). Collectively, the above results demonstrate that PTEN inhibits BMI1 function independently of its PIP3 phosphatase activity.

**Figure 4:**
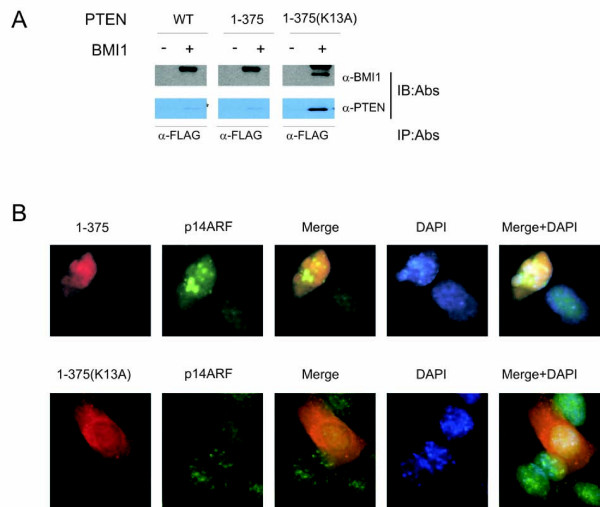
**Nuclear PTEN reduces BMI1 function**. (**A**) Interaction of nuclear PTEN with BMI1. Chimpanzee *PTEN *and the indicated mutants *PTEN/1-375 *and *PTEN/1-375(K13A) *were transfected without and with FLAG-tagged *BMI1 *in 293T cells, followed by immunoprecipitation of BMI1 using an anti-FLAG (α-FLAG) and then immunobloted (IB) with the indicated antibodies. Control IgG did not precipitate either BMI1 or PTEN (data not shown). (**B**) Nuclear PTEN inhibits BMI1 function. DU145 cells were transiently expressed with *PTEN/1-375 *(top panel) or *PTEN/1-375(K13A) *(bottom panel). Cells were then double IF stained for ectopic PTEN mutants using an anti-HA antibody (red) or endogenous p14^ARF ^(green). Nuclei were counter-stained with DAPI (blue). More than 200 transfected cells were randomly counted. Typical images of 1-375 and 1-375(K13A) were shown and the related quantification was discussed (see Discussion for details).

Since PTEN interacts with BMI1 exclusively in the nucleus (Fig 1), we examined the impact of nuclear and cytosolic PTEN mutants on BMI1's function. It has been shown that the Chimpanzee PTEN fragments 1-375 and 1-375/K13A resided largely in the nucleus and cytosol, respectively [37]. Dr. Pulido (Spain) kindly provided us these PTEN mutants together with Chimpanzee PTEN. When co-expressed in 293T cells, PTEN, 1-375, and 1-375/K13A were all co-precipitated via BMI1 (Fig 4A). While 1-375/K13A largely localizes in the cytosol, a small proportion of it remains in the nucleus [37] (Fig 4B). This remaining PTEN 1-375/K13A may contribute to the co-immunoprecipitation of 1-375/K13A via BMI1 (Fig 4A) (see Discussion for details). However, in comparison to 1-375/K13A, 1-375 displayed enhanced activity in up-regulation of endogenous p14^ARF ^when transiently transfected into DU145 cells (Fig 4B) (see Discussion for details). The up-regulated p14^ARF ^shows the typical pattern of nucleolar distribution (Fig 4B) [38], suggesting that the enhanced p14^ARF ^was functional. Taken together, these observations support the concept that nuclear PTEN reduces BMI1 function.

**Figure 5:**
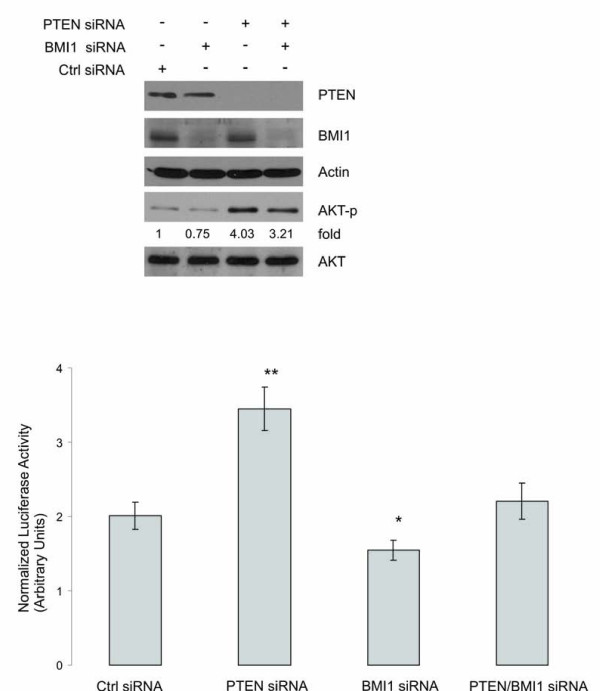
**PTEN reduces hTERT promoter activity via inhibiting BMI1 function**. DU145 cells were transfected with 100 nM PTEN siRNA, BMI1 siRNA, PTEN siRNA plus BMI1 siRNA, and the respective control (Ctrl) siRNA (Dharmcon) using LipofectAMINE2000 (Invitrogene) for 48 hours following our published procedure [52]. The expression of individual proteins was examined by western blot using specific antibodies (top panel). These cells were transfected with a pGL3-hTERTmin-Luc reporter, a lacZ vector, and plus the indicated siRNAs for 48 hours. Luciferase activities were determined and normalized against the respective lacZ activity. Experiments were carried out in triplicate and were repeated three times. Average data of these independent experiments is shown. Luciferase activities in PTEN siRNA (*p *< 0.001) and BMI1 siRNA (*p *< 0.05) cells are significantly different from that in Ctrl siRNA cells (bottom panel).

To further consolidate the concept that PTEN reduces BMI1 function, we knocked-down PTEN and BMI1 in DU145 cells using specific siRNAs (Fig 5, top panel). As expected, knockdown of PTEN activated AKT (Fig 5, top panel) and knockdown of BMI1 increased p14^ARF ^and p16^INK4A ^expression (data not shown). Consistent with BMI1 increasing hTERT promoter activity (Fig 3C), we observed that knockdown of BMI1 reduced hTERT promoter activity to approximately 77% of that observed in control siRNA treated DU145 cells (Fig 5, bottom panel). Furthermore, knockdown of PTEN significantly enhanced hTERT promoter activity in DU145 cells (Fig 5, bottom panel) and this occurs only in cells expressing endogenous BMI1 and not in cells whose BMI1 was concomitantly knocked-down (Fig 5, bottom panel), which demonstrates that endogenous PTEN reduces hTERT promoter activity via inhibiting endogenous BMI1 function.

### BMI1 reduces PTEN function

**Figure 6:**
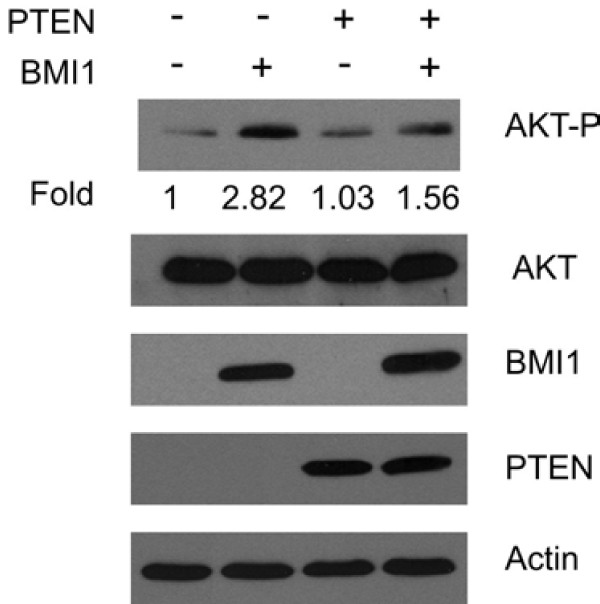
**BMI1 reduces PTEN's ability to inactivate the PI3K-AKT pathway**. DU145 cells were infected by PTEN, BMI1, or the respective empty retrovirus (-) as indicated. Cells were selected in the respective antibiotics for a few days to achieve 100% infection. The expression of AKT phosphorylation (AKT-P), total AKT (AKT), ectopic BMI1, ectopic PTEN, and actin was determined by western blot using the respective antibodies. The relative levels of AKT-P were quantified.

It is well established that PTEN antagonizes the activity of PI3K via dephosphorylation of membrane-bound PIP3. This inhibition is dependent on recruiting cytosolic PTEN to the plasma membrane. Since BMI1 binds PTEN in the nucleus, high levels of BMI1 may attenuate PTEN's ability to inhibit the PI3K-AKT pathway by sequestering PTEN in the nucleus. To test this possibility, we infected DU145 cells with a retrovirus expressing BMI1, PTEN, or both BMI1 and PTEN (Fig 6). BMI1 overexpression indeed enhanced AKT activation (Fig 6). While overexpression of PTEN alone did not affect AKT activation in DU145 cells, which is consistent with other publications [39,40], ectopic PTEN reversed the increase in AKT activation observed in DU145 cells overexpressing BMI1 (Fig 6). As ectopic PTEN alone does not directly affect the PI3K-AKT pathway (Fig 6), ectopic PTEN may indirectly affect the PI3K-AKT pathway by interacting with ectopic BMI1. This possibility is further supported by the observation that knockdown of BMI1 alone or in combination with knockdown of PTEN slightly reduced AKT activation (Fig 5, top panel, comparing AKT phosphorylation in BMI1 siRNA and PTEN siRNA/BMI1 siRNA lanes with that in Ctrl siRNA and PTEN siRNA lanes, respectively).

### PTEN appears to co-localize with BMI1 in primary prostate cancer

**Figure 7:**
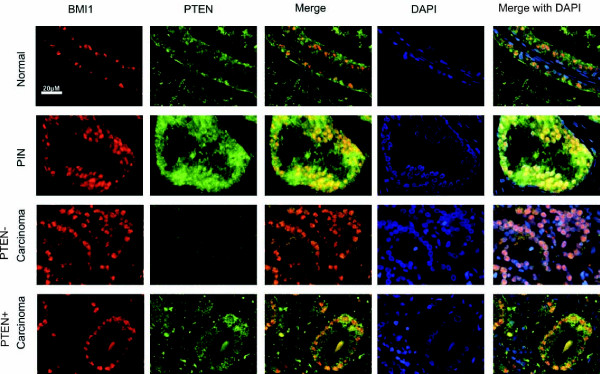
**Co-localization between PTEN and BMI1 in primary prostate cancer tissues**. Double IF staining for BMI1 (red) and PTEN (green) in normal prostatic gland (normal), PIN, PTEN-negative carcinoma, and PTEN-positive carcinoma. Nuclei were counter-stained with DAPI (blue). Images were captured with a confocal microscope. Scale bar represents 20 μM.

The fact that PTEN binds BMI1 and reduces BMI1 function in cultured prostate cancer cells prompted us to examine whether PTEN co-localizes with BMI1 in primary prostate cancer. While PTEN marginally co-localizes with BMI1 in normal prostate epithelial cells, PTEN extensively co-localizes with BMI1 in PIN and in PTEN positive (but not negative) prostate carcinoma (Fig 7), which further demonstrates the specificity of the anti-PTEN antibody used. We performed double immunofluorescent (IF) staining for PTEN and BMI1 in 42 primary prostate cancer specimens. By taking advantage of the heterogeneous nature of primary prostate cancer specimens [41], we were able to locate normal prostate glands, PIN, and carcinoma within each specimen. One thousand cells were randomly counted within individual tissues (normal prostate glands, PINs, and carcinomas) for each specimen in our sample set. Approximately 56% of prostate carcinomas express PTEN (data not shown), a number that is consistent with previous publications [29]. While only 2.4% of epithelial cells from normal prostate glands show low levels of co-localization between PTEN and BMI1 (Fig 7, Table 1), 37.6% and 18.5% of PIN and carcinoma cells display extensive co-localization between PTEN and BMI1 (Fig 7, Table 1). Interestingly, while PTEN stays largely outside of the nucleus in the epithelial cells of normal prostate glands, a significant increase in nuclear PTEN is observed in PINs and PTEN-positive carcinoma (Fig 7). This may be attributable to the observed increases in the co-localization between PTEN and BMI1 in PINs and PTEN-positive prostate carcinoma in comparison to normal prostate epithelium (Fig 7, Table 1). Taken together, the above observations suggest that nuclear PTEN plays an important role in inhibiting BMI1 function during prostate tumorigenesis.

**Table 1:** Co-localization between PTEN and BMI1 in primary prostate cancer

**PTEN and BMI1 co-localization (%)**		***p*-value**	
Normal	2.4 ± 9.5	Normal versus PIN	<0.001
PIN	37.6 ± 7.5	Normal versus Carcinoma	0.024
Carcinoma	18.5 ± 5.5	PIN versus Carcinoma	0.009

Statistical analysis (2-tails) was performed using GraphPad 4.0 for Windows

## Discussion

While it is known that BMI1 promotes tumorigenesis, at least in part, by suppressing p16^INK4A ^and p14^ARF ^expression, it remains to be determined what mechanisms regulate BMI1-mediated oncogenic activities. We demonstrate here that one of these mechanisms is PTEN-mediated attenuation of BMI1 function.

PTEN physically associates with BMI1 in cultured prostate cancer cells and appears to co-localize with BMI1 in primary prostate cancer. It was observed that PTEN co-localizes with BMI1 more extensively in PINs compared to both carcinomas and normal prostate glands (Fig 7; Table 1). As high grade PINs are pre-cancerous lesions, the above observation supports the concept that PTEN inhibits (or plays a surveillant role to) BMI1's oncogenic activities during prostate tumorigenesis and that escape from PTEN's suppression (or surveillance) enables BMI1 to promote prostate cancer progression. This is consistent with the observed increases in *BMI1 *mRNA [18] and BMI1 protein [32] levels during prostate tumorigenesis. Through its interaction with BMI1, PTEN inhibits BMI1's function.

The above conclusion is further supported by the observations that PTEN binds to BMI1 in the nucleus (Fig 1) and that a nuclear PTEN mutant (1-375) was more potent in inhibiting BMI1-mediated reduction of p14^ARF ^than its cytosolic counterpart (PTEN mutant 1-375/K13A) (Fig 4B). However, 1-375/K13A was efficiently immunoprecipitated through BMI1 (Fig 4A). This could be attributable to two factors, 1) 1-375/K13A is not an exclusive cytosolic protein [37] (Fig 4B) and 2) substitution of K13 with alanine may alter the protein conformation, which could increase its affinity to BMI1. Therefore, although there is far less 1-375/K13A in the nucleus, an increase in its binding affinity to BMI1 may allow 1-375/K13A to be efficiently co-immunoprecipitated via BMI1 (Fig 4A). Alternatively, the effective co-immunoprecipitation of 1-375/K13A via BMI1 might be an artifact caused by the cell lysate preparation, which allowed cytosolic 1-375/K13A to interact with nuclear BMI1. The association of nuclear 1-375/K13A (although a minor population) with BMI1 is consistent with our observation that approximately 11% of 1-375/K13A transfected cells displayed upregulation of endogenous p14^ARF^, while approximately 30% of 1-375 transfected DU145 cells showed p14^ARF ^upregulation. Taken together, these results support the notion that nuclear PTEN reduces BMI1 function.

While it is well documented that PTEN suppresses tumorigenesis via its PIP3 phosphatase activity at the plasma membrane, the potential function of nuclear PTEN is less clear. As PTEN appears to co-localize with BMI1 in the nucleus, our work suggests that nuclear PTEN inhibits BMI1 function, which is independent of PTEN's phosphatase activity. This is consistent with recent reports showing that nuclear PTEN maintains chromosome stability independently of its phosphatase activity [42] as well as induces G1 and G2 arrest in breast cancer and melanoma cells [31,43]. PTEN may regulate cell cycle progression via modulating p21^CIP1 ^[44]. Importantly, loss of nuclear PTEN was observed to associate with the tumor progression of melanoma and colorectal cancer [45,46]. Interestingly, the nuclear PTEN maintains chromosome stability via a C2 domain-mediated interaction with centromeres [42]. This agrees well with our finding that nuclear PTEN binds to BMI1 through the N-terminal 101 residues of its C2 domain (Fig 2B-D). Germline mutations of *PTEN *have been well documented to cause *PTEN*-deficient syndromes, such as the *PTEN *hamartoma tumor syndrome (PHTS) [47]. Two PHTS-associated hotspot mutations, R233X and R235X [47], lie in our defined BMI1 binding region (residues 186-286) (Fig 2B-D). Based on the above observations, it is tempting to propose that the C2 domain may play an important role in the tumor suppression function of nuclear PTEN and that R233 and R235 residues are functionally important to PTEN's interaction with BMI1.

The interaction with BMI1 modestly reduces PTEN's ability to inhibit the PI3K/AKT pathway (Fig 6). This can be attributed to BMI1 overexpression and the potential BMI1-mediated sequestration of PTEN in the nucleus. Whether changes in BMI1 expression *in vivo *can activate the PI3K-AKT pathway requires further investigation, as knockdown of BMI1 only slightly reduced AKT activation (Fig 5, top panel). This cautious interpretation is supported by our observations that **1) **ectopic expression of BMI1 in DU145 cells did not affect cell cycle distribution [G1 (58.18%)-S (34.44%)-G2/M (7.37%) for empty vector DU145 cells versus G1 (59.32%)-S (31.25%)-G2/M (9.52%) for BMI1-overexpressing DU145 cells] and **2) **ectopic BMI1 did not reduce ectopic PTEN-mediated growth inhibition of LNCaP and U87 cells (data not shown). Both of these events require PTEN's PIP3 phosphatase activity. BMI1 may also affect PTEN's nuclear functions. An intriguing possibility is that BMI1 may interfere with PTEN's ability to maintain chromosome stability, which adds an additional mechanism by which BMI1 can promote tumorigenesis. The increased co-localization of BMI1 and PTEN observed in PIN lesions and PTEN-positive prostate carcinomas compared to normal prostate epithelium supports this hypothesis (Table 1).

As PTEN is inactivated in human cancers [48], loss of PTEN function may release its inhibition on BMI1 during tumorigenesis. This would facilitate a role of BMI1 in promoting cancer stem cells, which is in line with the reported up-regulation of genes involved in stem cell self-renewal in hTERT-immortalized human cells [49]. Conversely, BMI1 may also inhibit PTEN function. It has recently been reported by van Lohuizen and his colleagues that BMI1 transgenic mice produce PINs in the prostate (see the meeting report of the CNIO Cancer Conference on Stem Cell and Cancer held between Feb 23-25, 2009 in Madrid, Spain) [50], which closely resembles the pathology observed in the prostate of PTEN^+/- ^mice [51]. Although the underlying mechanism regulating the interaction between PTEN and BMI1 is currently unknown, the physiological and functional association between these two proteins will certainly be an exciting avenue and warrants further investigation.

## Conclusion

This research demonstrates for the first time that nuclear PTEN reduces BMI1 function via an interaction with BMI1. PTEN inhibits BMI1 function independently of its phosphatase activity. This is different from PTEN-mediated inhibition of the PI3K/AKT pathway, which requires PTEN's PIP3 phosphatase activity. These results together with research on nuclear PTEN reported by other groups [23,31,43,45,46,49] indicate that nuclear PTEN represses tumorigenesis via multiple mechanisms. As PTEN plays a role in maintaining genome stability, our results suggest that by binding to PTEN, BMI1 may induce genome instability, which in turn promotes tumorigenesis.

## Competing interests

The authors declare that they have no competing interests.

## Authors' contributions

CF determined the interaction between PTEN and BMI1, PTEN-mediated inhibition of BMI1 function, and the relationship between PTEN and BMI1 in primary prostate cancer. LH characterized the interaction between PTEN and BMI1, including siRNA-mediated knockdown of PTEN and BMI1. AK collected primary prostate cancer and organized patient's information. APR examined the potential connection between PI3K and BMI1. JDM confirmed the interaction between PTEN and BMI1. JCC examined the pathologies associated with primary prostate cancer tissues. DT designed and supervised the experiments as well as finalized the manuscript.
